# Effect analysis of a virtual simulation experimental platform in teaching pulpotomy

**DOI:** 10.1186/s12909-022-03836-3

**Published:** 2022-11-07

**Authors:** Jiaxuan Lu, Xin Yang, Wei Zhao, Jiacheng Lin

**Affiliations:** Guanghua School of Stomatology, Hospital of Stomatology, Guangdong Provincial Key Laboratory of Stomatology, Sun Yat-Sen University, No.56 Lingyuan West Road, Guangzhou, 510055 China

**Keywords:** Virtual simulation experimental teaching, Virtual simulation experimental teaching platform, Experimental teaching, Pulpotomy, Pediatric dentistry

## Abstract

**Background:**

The experimental teaching of pediatric dentistry is a bridge between theoretical study and clinical practice, and virtual simulation technology provides a new method of instruction.

**Methods:**

We built an experimental teaching platform using virtual simulation technology for vital pulpotomy that includes learning and examination modes. A total of 199 students majoring in stomatology in the fourth year at Sun Yat-Sen University were randomly divided into a control group (conventional teaching mode) and an experimental group (virtual simulation experimental teaching model). The teaching effect was evaluated by theoretical and experimental examination.

**Results:**

We found that both the theoretical and experimental scores of the experimental group were higher than those of the control group, and the theoretical scores of the experimental group after exposure to the virtual simulation experimental teaching platform were also higher than those before the class, with significant differences (*P* < 0.05). Feedback from the experimental group after the class indicated that the platform reinforced their theoretical knowledge and greatly improved their mastery of operational skills.

**Conclusions:**

The application of a virtual simulation experimental teaching platform can effectively improve the teaching of pulpotomy.

## Background

With the popularization and development of modern educational technology, virtual simulation experimental teaching has become a new method of instruction. A characteristic of virtual simulation experimental teaching is that the operator can interact with a three-dimensional virtual reality environment generated by the computer [1–3]. Through the interaction between participants and the virtual simulation environment, and with the help of people’s own perceptions and cognitive abilities, various spatial and logical information contained in the virtual environment can be understood in a comprehensive way.

Virtual simulation experimental teaching is one of the reforms in higher education and is an important part of an experimental teaching demonstration center, the product of combining the discipline and information technology [4–6]. It focuses on the construction of networked experimental teaching resources to continuously promote the development of experimental teaching in colleges and universities [4–7].

In 2013, the Ministry of Education of the People’s Republic of China constructed a national virtual simulation experimental teaching center [8]. Virtual simulation technology is now being applied more frequently in the field of stomatology. Stomatology is a discipline that emphasizes strong practical ability and good comprehension. Wang et al. [9] used the Simodont virtual simulation system in cariology experimental teaching and found that it helps to improve students’ operational skills. Wang et al. [10] used the virtual technique in fixed denture teaching to effectively convey the abstract three-dimensional concept to dental students and enhance students’ three-dimensional perception of dental repair. Zhong et al. [11] combined the oral virtual simulation experimental teaching platform with a digital section on oral histopathology and found that virtual simulation experimental teaching could effectively stimulate students’ interest in learning, thus improving the teaching results.

However, there has not been any report about the application of a virtual simulation experimental platform in teaching pediatric stomatology. Pediatric dentistry is an important part of stomatology, and it covers a full range of oral diagnosis and treatment and preventive measures for children of a certain age [12–14]. The teaching of pediatric dentistry consists of three curriculum stages: theoretical study, experimental teaching, and clinical practice training.

Experimental teaching is the link between theoretical teaching and clinical practice. The existing experimental teaching of pediatric dentistry in China mainly covers aspects such as dental caries fillings, pulp resection, and root canal therapy. However, due to constraints of costs, laboratory management, and human and material resources, students cannot practice repeatedly to master the experimental contents immediately, and it is naturally impossible for them to acquire the corresponding technical knowledge before entering the clinic. In particular, a model of young permanent teeth with open apices is still lacking for projects such as young permanent tooth pulpotomy, apexification, and pulp regeneration therapy; therefore, students cannot perform relevant exercises in the classroom.

Endodontic treatment is an important part in teaching pediatric dentistry. Pulpotomy is a minimally invasive endodontic treatment concept that has been vigorously advocated in recent years and has received much attention, as it effectively makes up for the limitations of traditional endodontic treatment[15–17]. However, this technique is very demanding to perform, and the failure rate among students is high. Once this technique fails, the affected teeth develop an irreversible damage.

To resolve the difficulties in experimental teaching, our hospital (Guanghua School of Stomatology, Hospital of Stomatology, Sun Yat-sen University) selected pulpotomy, a common treatment in pediatric dentistry, and independently developed a virtual simulation experimental platform for teaching pulpotomy so that students could master the examination, diagnosis, treatment plan, and the specific operative steps of pulpotomy (www.ilab-x.com/details?id=2852&isView=true).

We investigated whether the application of a virtual simulation experimental platform for teaching pulpotomy can effectively improve the teaching quality in our school (Guanghua School of Stomatology, Sun Yat-sen University).

## Methods

### The characteristics of a virtual simulation experimental platform for teaching pulpotomy

**Fig. 1:**
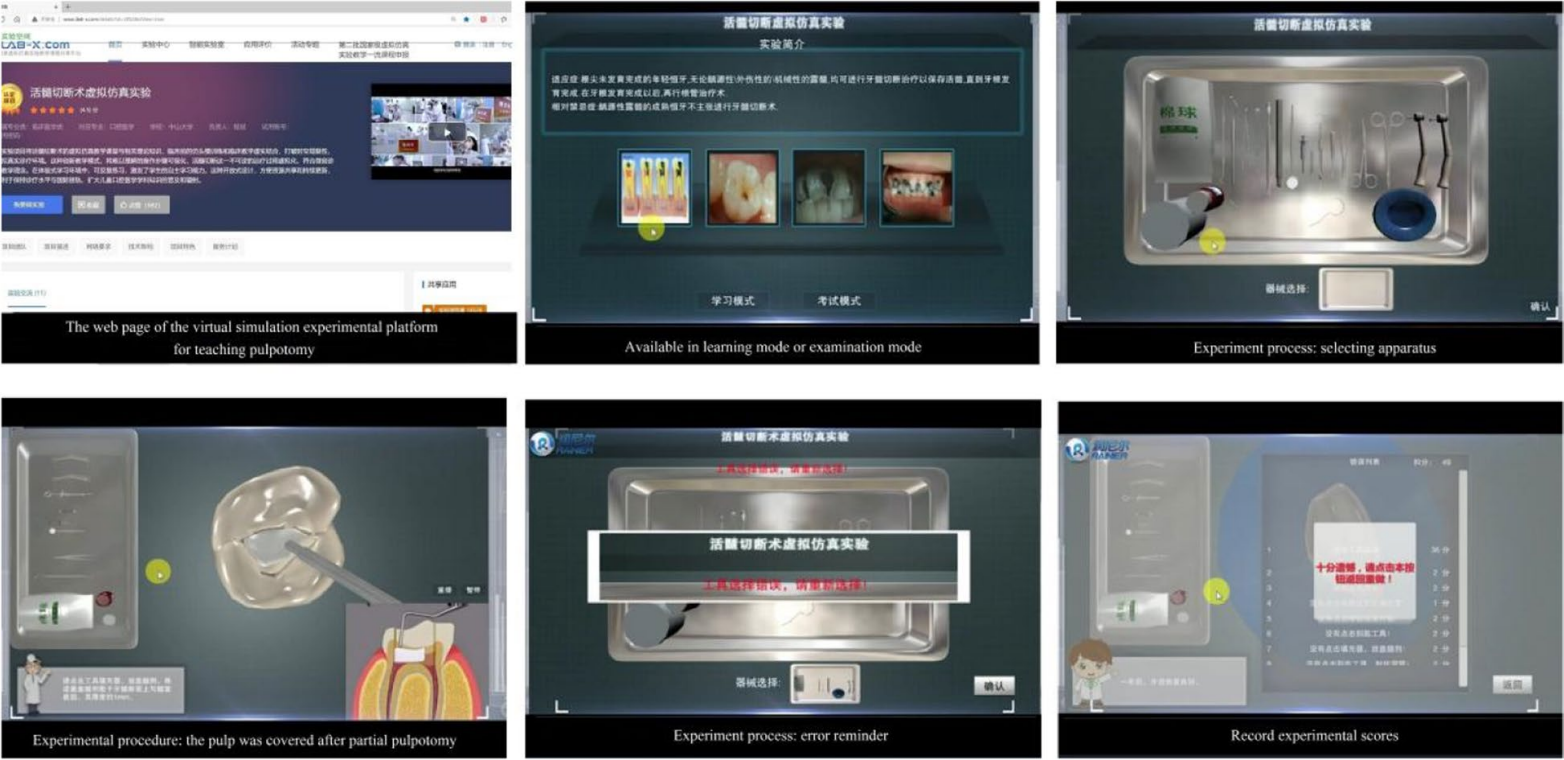
The Virtual Simulation Experimental Platform for Teaching Pulpotomy

This experimental platform is supported by Beijing Rainier Network Technology Co., Ltd. (Beijing, China; www.rainier.et.cn). The platform (www.ilab-x.com/details?id=2852&isView=true) is based on computer simulation, multimedia, and network technologies and uses a service-oriented B/S architecture design, including two modes of learning and examination (Fig. 1).

The design of the virtual environment, including the layout of the clinic and the materials and equipment for clinical application, were based on the environment in our hospital.

The experiment simulated the whole process of clinical reception, examination, diagnosis, and treatment of young permanent teeth after trauma and deciduous teeth with reversible pulpitis [18].

In the learning mode, users can complete the whole process of simulated case reception, examination, diagnosis, and pulpotomy of primary teeth and young permanent teeth step-by-step in a simulated clinical environment according to the prompts.

In the examination mode, the contents and standards of objective evaluation are presented as multiple-choice questions according to the key points that need to be mastered in clinical practice. Ten key points of clinical technology, such as indication selection and aseptic operation, are covered and corresponding scores are given. At the same time, the examination mode can be repeated and human–computer interactions analyzed retrospectively.

In addition, teachers can use the interactive aspect of network learning to track and understand students’ learning time, progress, and mastery of the material to promote students’ learning and improve teaching effectiveness. Moreover, to better assist students in learning, the platform also includes a film of the complete process of standard pulpotomy for students’ reference.

Not only students in Sun Yat-sen University but also users outside the university can visit and study. Users can access the platform for an unlimited amount of time.

### The research process

**Fig. 2:**
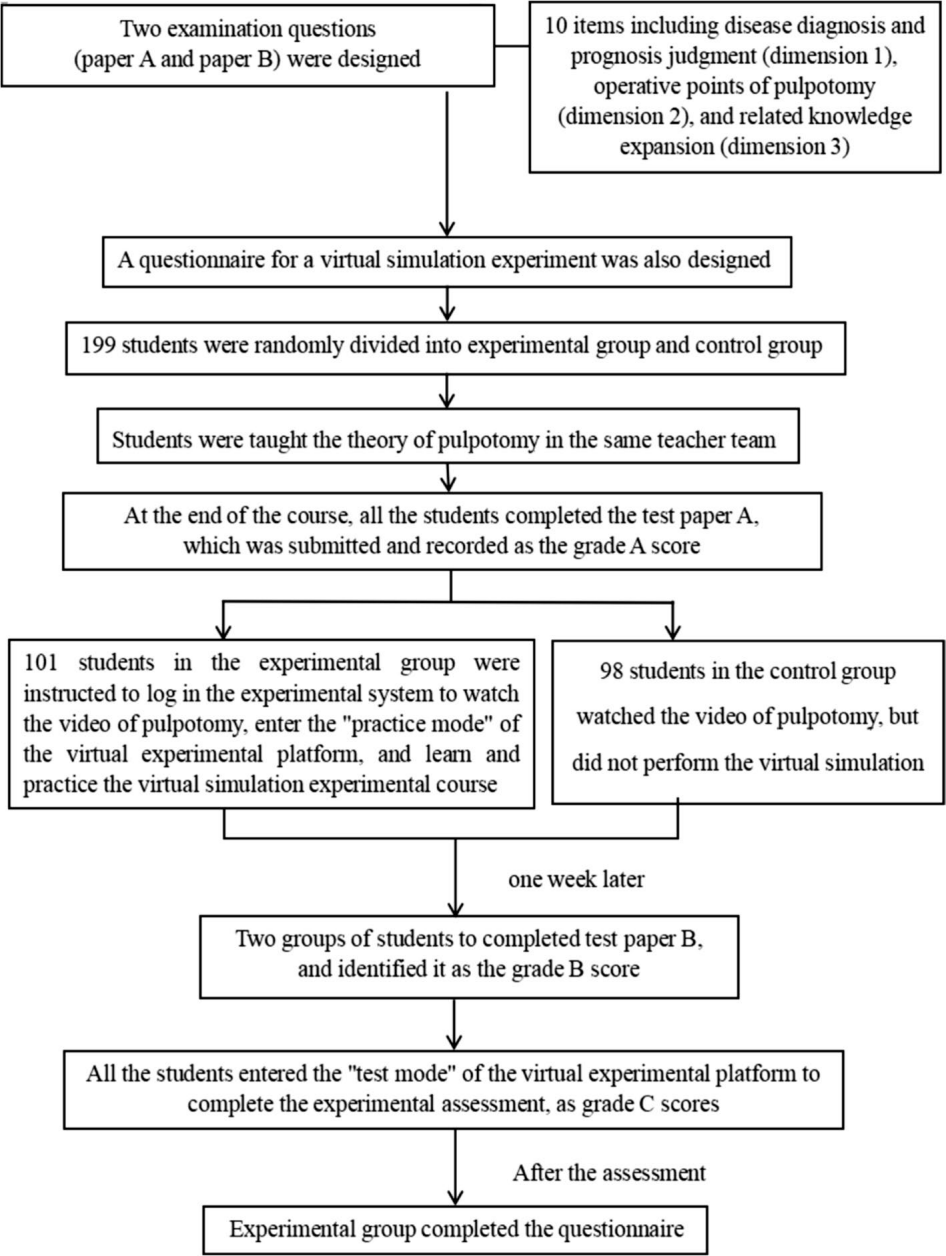
Design of the research study

In total, 199 students in the fourth year and majoring in stomatology at Guanghua School of Stomatology, Sun Yat-Sen University from May 2019 to December 2020 were randomly divided into a control group (received the conventional teaching mode, including theoretical teaching, watching pulpotomy teaching videos before the experimental class (without virtual simulation experimental platform training), experimental training, clinical probation, and clinical practice training) and experimental group (received the virtual simulation experimental teaching model, including theoretical teaching, watching pulpotomy teaching videos before the experimental class, virtual simulation experimental platform training, experimental training, clinical probation, and clinical practice training). The theoretical course and experimental training are taught by the same teacher respectively. There were 101 and 98 students in the experimental and control groups, respectively. Both groups completed 2 teaching hours of theoretical teaching, 2 teaching hours of watching pulpotomy teaching videos before the experimental class, and 2 teaching hours of experimental training. The experimental group completed virtual simulation experimental platform training after classes. The design of this research study is shown in Fig. 2.

Two objective assessment examination questions (paper A and paper B) were designed, including disease diagnosis and prognostic judgment (dimension 1), operative points of pulpotomy (dimension 2), and related knowledge expansion (dimension 3). There were 10 items in total in three dimensions. A questionnaire for a virtual simulation experiment was also designed.

At the end of the course, both the experimental and control groups completed test paper A, which was submitted and recorded as the grade A score.

Students in the experimental group were instructed to log into the experimental system to watch the pulpotomy video, enter the “practice mode” of the virtual experimental platform, and learn and practice the virtual simulation experimental course. In the control group, the students logged into the platform and watched the pulpotomy video but did not enter the “practice mode” of the experimental platform to perform the virtual simulation experiment.

According to the Ebbinghaus Forgetting Curve [19], the two groups of students completed test paper B a week later, and it was recorded as the grade B score. Then, they entered the “test mode” of the virtual experimental platform to complete the experimental assessment. The score data of the two groups of students in the “test mode” of the virtual experimental platform were designated as grade C scores. After the assessment, the experimental group completed the questionnaire. All the examination questions and questionnaires were completed in the class, with an effective recovery rate of 100%.

### Statistical analysis

The grade A, B, and C scores of the two groups of students were entered using SPSS 20.0 software (SPSS Inc., Chicago, Illinois, USA). The data are shown as means ± standard deviations. Statistical analyses were performed using one-way analysis of variance, and the t-test was used for multiple comparisons. *P* < 0.05 was considered statistically significant.

## Results

### Comparison of the grades between the two groups

The average grade A score of the experimental group was slightly lower than that of the control group, but there was no statistically significant difference between them (Table 1), confirming that the two groups of students were at the same baseline before the experiment.

**Table 1:** Comparison of the grade A scores

Grade A	Experimental Group (*n* = 101)	Control Group (*n* = 98)	*t*	*P*-value
Total points	7.28 ± 1.49	7.84 ± 1.37	-1.383	0.173
Dimension 1	3.32 ± 0.75	3.36 ± 0.70	-0.195	0.846
Dimension 2	2.32 ± 0.80	2.80 ± 1.00	-1.872	0.067
Dimension 3	1.56 ± 0.51	1.68 ± 0.56	-0.797	0.429

^*^ *p* < 0.05, ** p < 0.01

After the training on the virtual simulation experimental platform in pulpotomy teaching, the grades B and C scores in the experimental group were significantly higher than those of the control group (Tables 2 and 3), mainly on the operative points of pulpotomy (dimension 2).

**Table 2:** Comparison of grade B scores

Grade B	Experimental Group (*n* = 101)	Control Group (*n* = 98)	*t*	*P*-value
Total points	8.16 ± 1.11	7.60 ± 0.87	1.993	0.032 *
Dimension 1	3.28 ± 0.54	3.44 ± 0.51	-1.079	0.286
Dimension 2	3.44 ± 0.58	2.56 ± 0.65	5.036	0.000 **
Dimension 3	1.44 ± 0.51	1.64 ± 0.57	-1.313	0.195

^*^ *p* < 0.05, ** p < 0.01

**Table 3:** Comparison of grade C scores

		X ± S	*t*	*P*-value
Grade C	Experimental Group (*n* = 101)	85.0 ± 8.29	1.142	0.035 *
	Control Group (*n* = 98)	82.2 ± 9.02		

^*^ *p* < 0.05, ** p < 0.01

As shown in Table 4, the difference between grade A and grade B scores in the experimental group was statistically significant, indicating that scores after training were higher than those before training. However, there was no statistically significant difference between A and B scores in the control group (Table 5).

**Table 4:** Comparison of grade A and grade B scores in the experimental group

Variable	Grade A	Grade B	*t*	*P*-value
Total points	7.33 ± 1.40	8.20 ± 1.03	-4.419	0.000 **
Dmension 1	3.30 ± 0.75	3.27 ± 0.52	0.273	0.787
Dimension 2	2.37 ± 0.76	3.43 ± 0.57	-9.133	0.000 **
Dimension 3	1.60 ± 0.50	1.50 ± 0.51	0.902	0.375

^*^ *p* < 0.05, ** p < 0.01

**Table 5:** Comparison of grade A and grade B scores in the control group

Variable	Grade A	Grade B	*t*	*P*-value
Total points	7.83 ± 1.26	7.67 ± 0.84	0.926	0.362
Dmension 1	3.33 ± 0.66	3.43 ± 0.50	-0.648	0.522
Dimension 2	2.80 ± 0.96	2.57 ± 0.63	1.424	0.165
Dimension 3	1.70 ± 0.53	1.70 ± 0.53	0.000	1.000

^*^ *p* < 0.05, ** p < 0.01

### Feedback from the questionnaire in the experimental group

**Fig. 3:**
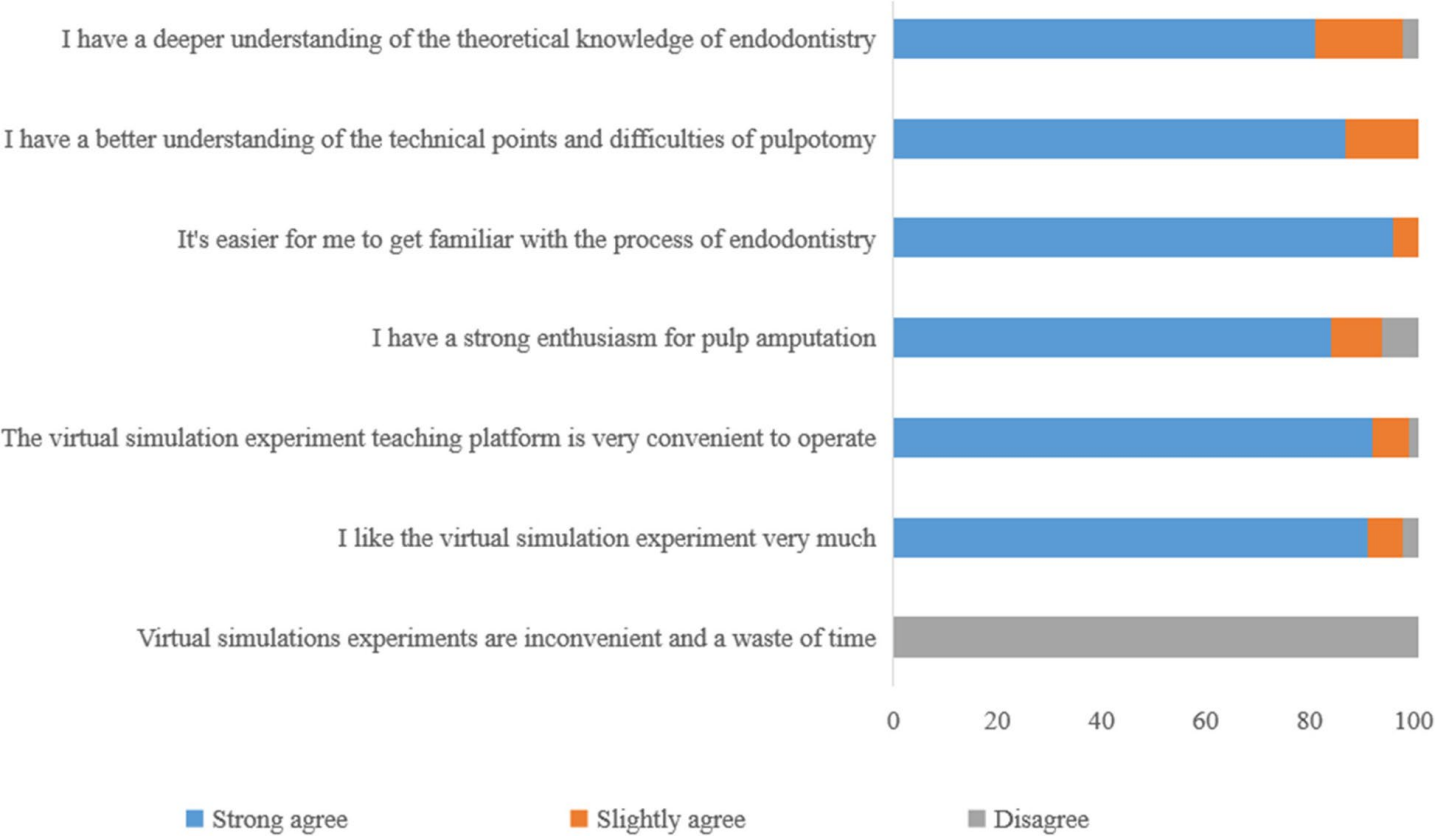
Questionnaire Results

Regarding the feedback from the questionnaire (Fig. 3), all students thought that the virtual simulation experimental teaching platform was very helpful for learning. Through the platform, it was easier to get familiar with the process of pulpotomy and master the difficult aspects and key points of the technology. More than 90% of the students liked this teaching method and had more enthusiasm for learning in the simulation condition.

**Fig. 4:**
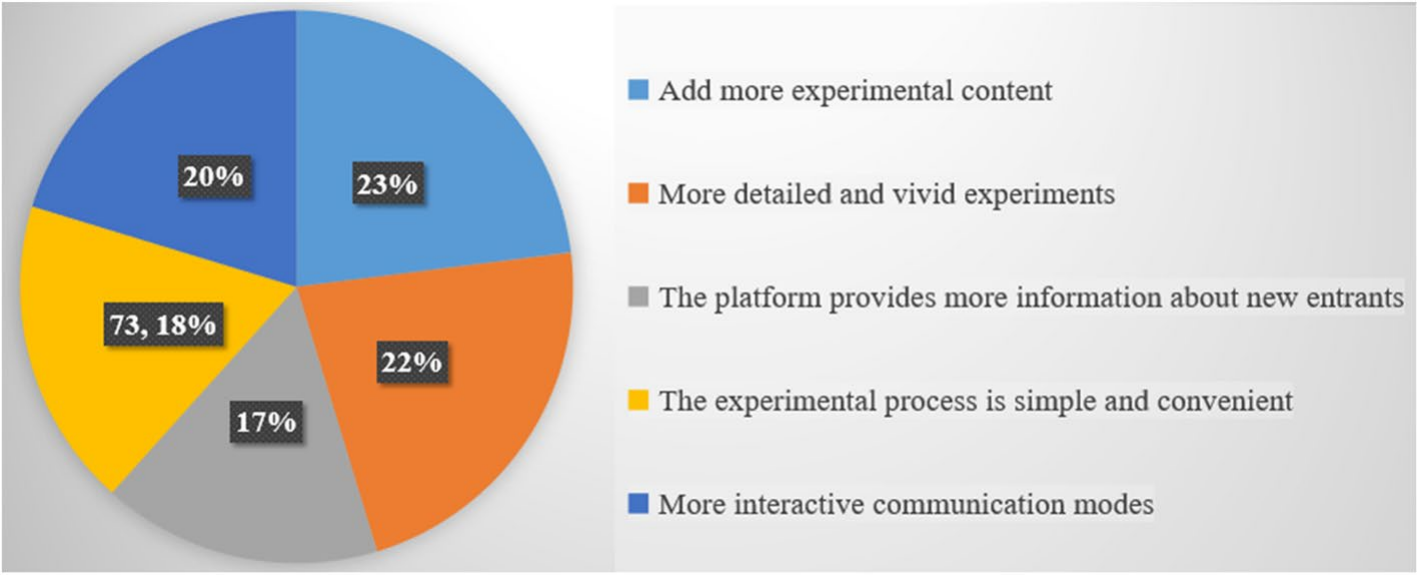
Outlook and Comments

Students also offered some opinions on the virtual simulation experimental teaching platform for pulp amputation (Fig. 4). They hoped that more experiments using in vitro teeth or dental models could be added to the virtual simulation experiment and that the experimental steps could be made more meticulous, virtual simulation effect more vivid, and the platform more interactive.

## Discussion

### The establishment of a virtual simulation experimental teaching platform is innovative

In China, patients are increasingly knowledgeable about procedures, and the medical conflict between doctors and patients is becoming increasingly prominent. Most children receiving oral treatment are unlikely to cooperate with the dentist, and parents often hope that their children can be treated by a skilled dentist. This requires students to master the operative technology before entering the clinic to establish a good foundation for future clinical work. The corresponding special experiments could not be simulated in the laboratory, including the surgical techniques mentioned in this study such as vital pulpotomy. However, opportunities to acquire clinical experience were uncertain, unavailable, or insufficient, leaving the students without enough opportunities to master the corresponding skills. The traditional teaching model, which mainly focuses on theory, cannot meet practical training requirements for dental students.

Pulp defense and self-repair is the biological basis of pulp repair and vital pulp preservation [20, 21]. Pulpotomy is a method of removing the crown pulp tissue under local anesthesia and covering the pulp wound with viable pulp to preserve the normal pulp tissue at the root [18]. It is difficult to perform pulpotomy in experimental teaching because the pulp condition of young permanent teeth cannot be simulated in the laboratory.

However, using virtual simulation technology, the abstract concepts and independent technical operations of the textbook can be transformed into intuitive and controllable simulation graphics and images, and teaching can be simplified [22–24]. Virtual simulation experimental teaching can solve many problems encountered in conventional offline teaching and become an important supplement to conventional teaching.

This study introduced virtual simulation technology. It can provide students with cases that simulate a doctor–patient environment, as well as repeatable virtual simulations of pulpotomy, by simulating the operation of pediatric dentistry departments and providing an interactive link for learning through virtual simulation teaching experiments.

The experimental survey showed that students using the virtual simulation experimental platform can learn repeatedly regardless of time and space constraints to become more familiar with the operative process and main technical points of pulpotomy. The platform can accurately illustrate the process of clinical diagnosis and treatment, and students can develop their skills in a noninvasive, risk-free, and repeatable way.

### The virtual simulation experimental teaching platform enhances teaching effectiveness and promotes the reform of experimental teaching

Previous studies have shown that virtual simulation teaching plays an auxiliary role in medical teaching, assessment, and autonomous learning [25–29]. The results of this study showed that after virtual simulation experimental training, the theoretical performance and operational skills were better in the experimental group than in the control group, and the use of the virtual simulation experimental system improved learning performance and teaching effectiveness, indicating that the teaching design is effective and should continue to be used and promoted.

In addition, the statistical differences in the theoretical examination mainly reflected differences in the pulp amputation operation dimension, and the operation examination scores were higher for the experimental group than for the control group. This indicates that the virtual simulation teaching platform is effective in improving practical skills in teaching pulpotomy. Compared with the self-study forms after class in the control group, students in the experimental group could preview lessons before class and review them after class on the platform at any time, which can improve the utilization of teaching resources and resource sharing. The platform has the advantages that the conventional teaching mode does not have. It can help students to develop their own thinking, promote the improvement of independent learning ability and practical ability, strengthen the mastery of relevant knowledge and skills, and encourage students to carry out active exploratory learning.

The virtual simulation teaching experimental system can record students’ personal learning trajectory. This project includes three parts: pre-class multimedia learning, virtual practice, and examination. The platform not only evaluates students’ mastery and proficiency of the technology through an interactive process but also has strict assessment and scoring standards. During and after the training, human–computer interaction can be retroactively analyzed according to the learning dynamic trajectory recorded by the platform to provide reference data for targeted optimization of teaching efficiency.

Compared with the traditional examination method, virtual simulation teaching is more timely and can measure training, assessment and make continuous improvements according to the feedback [30, 31]. Through the open platform, students can receive timely feedback from the instructor that helps learning and consolidation and interact with teachers online, which also significantly improves students’ learning initiative, enthusiasm, and efficiency. This is conducive to cultivating students’ independent learning.

Through the feedback on the questionnaire, all the students thought that the virtual simulation experiment platform was helpful for learning, and more than 90% of the students liked this learning method. Students also hoped that more educational content could be added to the virtual simulation teaching experimental platform. Through the combination of information technology and experimental teaching, the virtual simulation teaching experimental platform has developed an innovative teaching mode of “virtual simulation training and simulated head model experimental teaching.”

However, our study has some limitations. First, the virtual simulation teaching experimental platform can only deal with a specially appointed tooth and cannot simulate the anatomical morphology and root canal system of different teeth. Second, the clinical situation is often complex and changeable, but the virtual simulation teaching experimental platform is designed for specific clinical situation, which cannot really reproduce all the scenes completely.

In the future, we will continue to update and maintain the pulpotomy virtual simulation experimental teaching platform and promote the platform so that more dental students and dentists can benefit from it. On the other hand, virtual simulation experiment technology can be applied to more professional technology teaching, such as revascularization, replantation of traumatic teeth, etc.

## Conclusions

The combination of virtual simulation experimental teaching and real experiments can complement each other, which can significantly improve the effect of experimental teaching and is conducive for the sharing and optimization of educational resources. In addition, it can also mobilize students’ enthusiasm and initiative for participating in experimental teaching. Applying information technology to experimental teaching, establishing an open and networked virtual simulation experimental teaching system, and establishing a training model can effectively make up for the deficiency of traditional experimental teaching. Overall, we found that the virtual simulation platform can effectively improve the teaching quality of pulpotomy in our school.

## Data Availability

Because the account passwords of administrators and teachers cannot be shared, authors cannot deposit their datasets in publicly available repositories or render them publicly. All data and materials are available from the corresponding author upon request.
